# A pilot study of chlormequat in food and urine from adults in the United States from 2017 to 2023

**DOI:** 10.1038/s41370-024-00643-4

**Published:** 2024-02-15

**Authors:** Alexis M. Temkin, Sydney Evans, Demetri D. Spyropoulos, Olga V. Naidenko

**Affiliations:** 1 Environmental Working Group, Washington, DC USA; 2 Department of Pathology & Laboratory Medicine, Medical University of South Carolina,, Charleston, SC USA

**Keywords:** Biomonitoring, Pesticides, Endocrine disruptors, Children’s health

## Abstract

**Abstract:**

Chlormequat chloride is a plant growth regulator whose use on grain crops is on the rise in North America. Toxicological studies suggest that exposure to chlormequat can reduce fertility and harm the developing fetus at doses lower than those used by regulatory agencies to set allowable daily intake levels. Here we report, the presence of chlormequat in urine samples collected from people in the U.S., with detection frequencies of 69%, 74%, and 90% for samples collected in 2017, 2018–2022, and 2023, respectively. Chlormequat was detected at low concentrations in samples from 2017 through 2022, with a significant increase in concentrations for samples from 2023. We also observed high detection frequencies of chlormequat in oat-based foods. These findings and chlormequat toxicity data raise concerns about current exposure levels, and warrant more expansive toxicity testing, food monitoring, and epidemiological studies to assess health effects of chlormequat exposures in humans.

**Impact:**

- This study reports the detection of chlormequat, an agricultural chemical with developmental and reproductive toxicity, in the U.S. population and U.S. food supplies for the first time. While similar levels of the chemical were found in urine sampled from 2017 to 2022, markedly increased levels were found in samples from 2023. This work highlights the need for more expansive monitoring of chlormequat in U.S. foods and in human specimens, as well as toxicological and epidemiological study on chlormequat, as this chemical is an emerging contaminant with documented evidence of low-dose adverse health effects in animal studies.

## Introduction

Chlormequat chloride is an agricultural chemical first registered in the U.S. in 1962 as a plant growth regulator. Although currently only allowed for use on ornamental plants in the U.S, a 2018 decision by the U.S. Environmental Protection Agency (EPA) permitted the import of foods, primarily grains, treated with chlormequat [1]. In the European Union, the United Kingdom and Canada, chlormequat chloride is approved for use on food crops, primarily wheat, oats, and barley. Chlormequat acts to decrease stem height, thereby reducing the likelihood of crops bending over, which can make harvesting difficult. In the UK and European Union, chlormequat is often the most detected pesticide residue in grains and cereals, as documented by monitoring surveys spanning several years [2, 3].

Despite being approved for use on crops in Europe and parts of North America, chlormequat exhibits concerning toxicological properties, as documented in historical as well as more recently published laboratory animal studies. In the early 1980s, the impacts of chlormequat exposure on reproductive toxicity and fertility were first described by Danish pig farmers who observed reproductive declines in pigs raised on chlormequat treated grains [4]. These observations were later investigated in controlled laboratory experiments on pigs and mice, whereby female pigs fed chlormequat treated grain exhibited disrupted oestrus cycling and difficulty mating compared to animals on a control chlormequat-free diet [4]. Additionally, male mice exposed to chlormequat via diet or drinking water during development exhibited decreased fertilization capacity of sperm in vitro [5]. More recent reproductive toxicity studies on chlormequat show delayed onset of puberty, reduced sperm motility, decreased weights of male reproductive organs, and decreased testosterone levels in rats exposed during sensitive windows of development, including during pregnancy and early life [6–8]. Developmental toxicity studies also suggest that chlormequat exposure during pregnancy can dysregulate fetal growth and metabolism [9]. Other investigations did not find impacts of chlormequat on reproduction in female mice, male pigs, or a subsequent investigation of fertilization capacity in male mice developmentally and postnatally exposed to chlormequat [4, 10, 11]. Equivocal evidence in the toxicological literature on chlormequat may be due to differences in doses tested and outcomes measured as well as selection of model organism and the sex of laboratory animals. Consequently, further investigation is warranted.

Although recent toxicological studies indicate impacts of chlormequat on development, reproduction and the endocrine systems, the mechanism(s) by which these toxicological effects occur is not well understood. Some studies indicate that chlormequat likely does not act through well characterized mechanisms of endocrine disrupting chemicals, including through estrogen or androgen receptors and does not alter aromatase activity [12]. Other evidence suggests chlormequat may elicit adverse effects through altered steroid biosynthesis and induction of endoplasmic reticulum stress [8, 13].

While chlormequat is prevalent in commonly consumed foods in Europe, only a relatively small number of biomonitoring studies assessing human exposure to chlormequat exists. Chlormequat has a short half-life in the body of about 2–3 h, with a large fraction of experimental doses being excreted within 24 h based on studies involving human volunteers [14]. In general population samples from the United Kingdom and Sweden, chlormequat was detected in the urine of nearly 100 percent of study participants at frequencies and concentrations considerably higher than for metabolites of other pesticides such as chlorpyrifos, pyrethroids, thiabendazole, and mancozeb [14–17]. Studies in pigs indicate that chlormequat can also be detected in serum, as well as transferred into milk, but these matrices have not been investigated in humans or other laboratory animal models, although the potential presence of chemicals associated with reproductive harm in serum and milk has important implications for exposures during pregnancy and to infants [18].

In April 2018, the U.S. EPA published acceptable food tolerance levels for chlormequat chloride in imported oat, wheat, barley, and some animal products, which permitted the import of chlormequat into the U.S. food supply. The allowable levels were then increased for oats in 2020. To characterize the impact of these decisions regarding the emergence and prevalence of chlormequat in the U.S. adult population, this pilot study measured levels of chlormequat in urine of individuals from three geographic regions within the U.S. from 2017 to 2023, as well as levels of chlormequat in oat and wheat-based products purchased in the U.S. in 2022 and 2023.

## Materials and methods

### Human urine samples

To measure chlormequat in urine from people residing in the U.S., convenience samples from three geographical regions collected between 2017 and 2023 were used. Twenty-one urine samples, collected on an Institutional Review Board (IRB)-approved protocol in 2017 from consenting de-identified pregnant women at time of delivery, were obtained from the Medical University of South Carolina (MUSC, Charleston, South Carolina, USA). Samples were stored at 4 °C for up to 4 h prior to being aliquoted and frozen at −80 °C. Twenty-five adult urine samples, were purchased in November of 2022 from Lee Biosolutions, Inc (Maryland Heights, Missouri, USA), representing single time point samples collected from October 2017 through September 2022, provided from volunteers (13 male and 12 female) collected in Maryland Heights, Missouri. The samples were stored at −20 °C immediately after collection. Additionally, 50 urine samples, collected in June of 2023 from volunteers in Florida (25 male, 25 female), were purchased from BioIVT, LLC (Westbury, NY, USA). Samples were stored at 4 °C until all samples were collected prior to being aliquoted and frozen at −20 °C. The supplier companies obtained necessary IRB approval for work with human samples and obtained consent for collection of the samples. No personally identifiable information was provided with any of the samples that were tested. All samples were shipped frozen for analysis. Detailed sample information can be found in the Supplementary information, Table S1.

### Analytical methodology

Chlormequat was quantified in human urine samples by LC MS/MS at the HSE Science and Research Laboratory (Buxton, United Kingdom) following the methodology published by Lindh et al. 2011 with slight modifications [14]. Briefly, the samples were prepared by mixing 200 µL of unfiltered urine with 1.8 mL of 0.01 M ammonium acetate containing an internal standard. The samples were then extracted using HCX-Q columns conditioned with methanol, then 0.01 M ammonium acetate, washed with 0.01 M ammonium acetate, and eluted into methanol with 1% formic acid. Samples were then loaded onto a C18 LC column (Synergi 4 µ Hydro-RP 150 × 2 mm; Phenomenex, UK) and separated using an isocratic mobile phase consisting of 80:20 0.1% formic acid: methanol at a flow rate of 0.2 mL/min. Mass spectrometry selected reaction monitoring transitions are described in Lindh et al. 2011. The limit of detection was 0.1 µg/L, as reported in other studies [14].

Chlormequat concentrations in urine were reported as µmol chlormequat per mol creatinine and converted to µg chlormequat per g creatinine, as expressed in previous studies (multiplied by 1.08).

Oat (25 conventional and 8 organic) and wheat-based (9 conventional) food samples purchased at U.S. grocery stores in the Washington, DC metro area from June and August 2022, and February and May 2023 were tested for chlormequat by Anresco Laboratories, LLC (San Francisco, CA, USA). Samples were analyzed based on published methodologies with modifications [19]. The LOD/LOQ were set at 10/100 ppb and 3/40 ppb for oat samples from 2022 and for all wheat and oat samples from 2023 respectively. Detailed sample information can be found in the Supplementary information, Table S2.

### Statistical analysis

Urinary chlormequat concentrations were grouped together based on geographic location and collection year except for two samples from Maryland Heights, MO collected in 2017, that were grouped with the other 2017 samples from Charleston, SC. Samples that were below the limit of detection for chlormequat were treated as LOD divided by the square root of two. The data were not normally distributed, therefore a Kruskal-Wallis nonparametric test with Dunn’s multiple comparisons test was used to compare the medians between groups. All calculations were done in GraphPad Prism (Boston, MA).

## Results

**Fig. 1:**
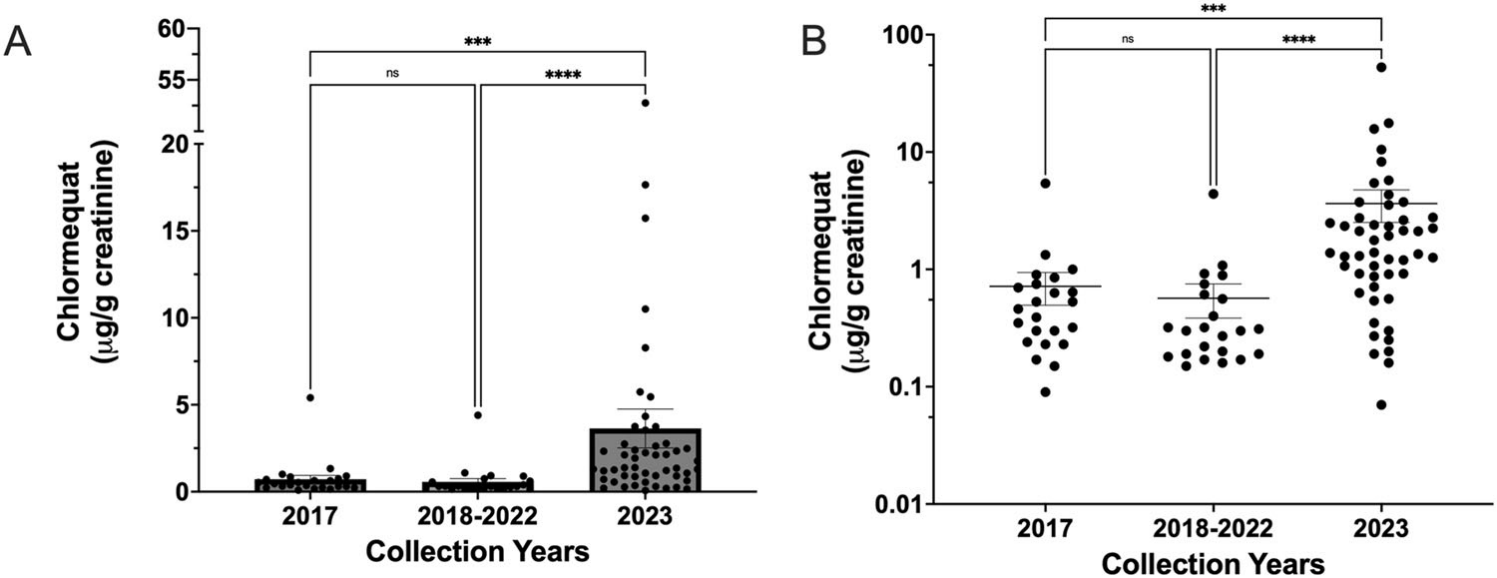
Chlormequat concentrations in urine from individuals residing in the U.S. at three time points. Chlormequat concentrations for each individual urine sample are represented by single dots with bars at the mean and error bars representing +/- standard error. Urinary concentrations of chlormequat are expressed as µg chlormequat per g creatinine on a linear scale (**A**) and log scale (**B**). Kruskal-Wallis nonparametric ANOVA with Dunn’s multiple comparisons test was used to test statistical significance (***p < 0.001, ****p < 0.0001).

Chlormequat was detected in 77 of 96, or 80% of all urine samples. Detection frequencies were higher in 2023 samples compared to 2017 and 2018 to 2022 samples with 16 of 23, or 69%, 17 of 23, or 74% and 45 of 50, or 90% of samples with detections, respectively (Table 1). The concentrations of chlormequat detected were comparable between the two groups before 2023, while the concentrations detected in 2023 samples were significantly higher than previous years samples (Fig. 1A, B). The concentrations among detectable samples ranged from 0.22 to 5.4, 0.11 to 4.3, and 0.27 to 52.8 µg chlormequat per g creatinine for 2017, 2018–2022, and 2023 samples, respectively. The median values from all samples were 0.46, 0.30, and 1.4 for 2017, 2018–2022, and 2023 samples, respectively (Table 1). These data indicate likely continuous exposure given the short half-life of chlormequat in vivo, with low levels from 2017 to 2022 and higher exposure levels in 2023.

**Table 1:** Comparison of urinary chlormequat concentrations across studies.

Study (Location)	Collection Year	Sample Number	Detection Frequency (N)	Range (*μ*g chlormequat per g creatinine)	Median	LOD (μg/L chlormequat)
Present (Florida, USA)	2023	50	90% (45)	ND - 52.8	1.4	0.1
Present (Missouri, USA)	2018–2022	23	74% (17)	ND - 4.3	0.3	0.1
Present (South Carolina, USA and Missouri, USA)^a^	2017	23	69% (16)	ND - 5.4	0.46	0.1
Noren et al. 2019 (Sweden)	2017	196	100% (196)	NA - 35.8	0.86	0.01
Galea et al. 2015 (United Kingdom)	2011–2012	140	~99% (NA)	NA - 388.2	15.1	0.6
Lindh et al. 2011 (Sweden)	2010	100	100% (100)	0.4–30.2	2.9	0.1

*NA* indicates value was not reported in the study, *ND* none detected.

^a^Two samples from Missouri were collected in 2017.

Food samples purchased in the U.S. from 2022 and 2023 show detectable levels of chlormequat in all but two of 25 conventional oat-based products, with concentrations ranging from non-detectable to 291 µg/kg, indicating a high prevalence of chlormequat in oats. Median levels were similar between samples collected in 2022 and 2023, at 90 and 114 µg/kg respectively. Only one sample out of eight organic oat-based products at detectable chlormequat at 17 µg/kg. We also observed low concentrations of chlormequat in two of nine wheat-based products tested, at 3.5 and 12.6 µg/kg (Table 2).

**Table 2:** Chlormequat in oat and wheat-based products purchased in U.S. grocery stores.

Food Type (Year)	Number of Samples Tested	Samples With Detections	Range	Median (μg/kg or ppb)	LOD/LOQ (μg/kg or ppb)
Conventional Oat-based products (May 2023)	12	11 (92%)	ND - 209	114	3/40
Conventional Oat-based products (June and August 2022)	13	12 (92%)	ND - 291	90	10/100
Organic Oat-based products (2023)^a^	8	1 (12.5%)	ND - 17	ND	3/40
Conventional Wheat-based products (February 2023)	9	2 (22%)	ND - 12.6	ND	3/40

*ND* none detected.

^a^One organic sample was purchased in 2022; individual sample results can be found in Table S2.

## Discussion

This is the first report of urinary chlormequat measurements in adults residing in the U.S., and any population outside of the United Kingdom and Sweden. A time-course of pesticide biomonitoring in over 1000 adolescents from Sweden documented 100 percent detection frequencies of chlormequat from 2000 to 2017. The median concentrations in 2017 were 0.86 µg chlormequat per g creatinine and appeared to decrease over time, the highest median level was 2.77 in 2009 [16]. In the UK from 2011 to 2012, biomonitoring detected much higher median concentrations at 15.1 µg chlormequat per g creatinine, and while these samples were collected from people residing in an agricultural area, there were no differences in exposure when levels were compared before and after chlormequat spray events [15]. Compared to these previous studies in Europe, the median levels measured in our study of U.S. samples from 2017 to 2022 were lower, while the median level in 2023 samples were comparable to samples from Sweden, and lower than UK samples (Table 1).

Differences in exposure between these different regions and time points likely reflects differences in agricultural practices and regulatory status of chlormequat, ultimately impacting chlormequat levels in the food supply. For instance, the significantly higher concentrations of chlormequat in the 2023 urine samples compared to earlier years may reflect the likely recent introduction of chlormequat into the U.S. food supply due to EPA regulatory action changes involving chlormequat, including establishing limits on chlormequat in food in 2018 and raising those limits for oats in 2020. These actions permitted import and sale of agricultural products that had been treated with chlormequat, for example from Canada. The time-lag between EPA regulatory changes and elevations in chlormequat concentrations found in the 2023 urine samples could be explained by several scenarios such as delays in adoption of agricultural practices that use chlormequat, delays in U.S. companies establishing trade agreements and exhausting old product stocks, and/or delays in individuals purchasing oats given the long expiration dates on oat products.

To determine if the concentrations observed in U.S. urine samples were reflective of potential dietary exposure to chlormequat, we measured chlormequat in oat and wheat-based food products purchased in the U.S. in 2022 and 2023. Oat products contained chlormequat more frequently than wheat products and the amount of chlormequat in different oat products varied, with a median level of 104 ppb, perhaps owing to sourcing from the U.S. versus Canada, potentially representing differences between products made with or without chlormequat-treated oats. Comparatively, in food samples from the United Kingdom, chlormequat is far more prevalent in wheat-based products such as bread in which 90% of samples collected in the UK between July and September 2022 had detectable levels of chlormequat with a median concentration of 60 ppb. Similarly, chlormequat was detected in 82% of UK oat samples from the same time with median concentrations of 1650 ppb, more than 15 times higher than in U.S. samples, which may explain the higher urinary concentrations observed in UK samples [20].

Our biomonitoring results indicate exposure to chlormequat before 2018, despite there being no established food tolerances set for chlormequat. Although there is no monitoring of chlormequat in food products in the U.S., and no historical data available for concentrations of chlormequat in foods sold in the U.S., we suspect these exposures were likely dietary given the short half-life of chlormequat. Additionally, natural formation of chlormequat from choline precursors in wheat products and egg powder has been shown to occur under high temperatures such as those used during food processing and production, resulting in chlormequat concentrations between 5 and 40 ng/g [21]. Our food testing results indicate chlormequat levels in some samples, including one organic oat-based product, were similar to those reported in the study of naturally forming chlormequat, while many other samples were higher. Thus, our pre-2023 levels observed in urine could be a result of dietary exposure to chlormequat from formation via food processing and production. And the 2023 levels observed could be due to a combination of dietary exposure to chlormequat from spontaneous formation as well as imported products agriculturally treated with chlormequat. Differences in chlormequat exposure in our samples could also be due to geographic locations, differing dietary patterns, or occupational exposure from uses of chlormequat in greenhouses and nurseries.

Our study indicates that a greater sample size and more diverse sampling of processed foods for chlormequat is needed to fully assess potential dietary sources of chlormequat in the low exposure individuals. Future studies that include the analysis of historical urine and food samples, dietary and occupational questionnaires, and continued monitoring of chlormequat in the U.S. food supply for both conventional and organic foods, as well as samples for biomonitoring would help elucidate the determinants of total chlormequat exposure in the U.S. population.

It remains to be determined whether chlormequat levels in U.S. urine and food samples may rise in the coming years. In the U.S., chlormequat is currently only allowed in imported oat and wheat products, but domestic agricultural uses on non-organic crops are currently under review by the U.S. EPA. It is possible that if such domestic uses were approved, combined with widespread adoption of agricultural practices that utilize chlormequat abroad and domestically, chlormequat levels would likely continue to increase in oats, wheat, and other grain foods, leading to higher levels of exposure for the U.S. general population.

Current chlormequat concentrations in urine from this study and others suggest that individual sample donors were exposed to chlormequat at levels several orders of magnitude below the reference dose (RfD) published by the U.S. EPA (0.05 mg/kg bw/day) and the acceptable daily intake (ADI) value published by the European Food Safety Authority (0.04 mg/kg bw/day). However, we note that published toxicological studies on chlormequat suggest reevaluation of these safety thresholds may be warranted. For instance, animals exposed to doses lower than the current RfD and ADI, of 0.024 and 0.0023 mg/kg bw/day in mice and pigs respectively, exhibited reduced fertility [4]. In another toxicological study, exposure during pregnancy at a dose equivalent to the No Observed Adverse Effect Level (NOAEL) of 5 mg/kg that was used to derive the U.S. EPA reference dose, caused altered fetal growth as well as metabolism and body composition in neonatal mice [9]. Additionally, the regulatory thresholds do not consider the adverse effects of mixtures of chemicals that may impact the reproductive system, which have been shown to cause additive or synergistic effects at doses lower than for individual chemical exposures [22], raising concerns about the potential health effects associated with current exposure levels, especially for individuals on the higher end of exposure in general populations of Europe and the U.S.

This pilot investigation into an emerging chemical exposure within the U.S. indicates that chlormequat chloride is present in the U.S. food supply, primarily in oat-based products, and is detectable in a majority of urine samples collected from nearly 100 individuals in the U.S., suggesting continuous exposure. Additionally, trends in these data suggest that exposure levels have increased and might continue to increase in the future. Given the toxicological concerns associated with chlormequat exposure in animal studies, and widespread exposure to the general population, in European countries, and now also likely in the U.S., monitoring of chlormequat in foods and people, in conjunction with epidemiological and animal studies, is urgently needed to understand the potential health harms of this agricultural chemical at environmentally relevant exposure levels, particularly during pregnancy.

### Supplementary information

Supplementary tables

## Data Availability

The data generated and analyzed in this study can be found within the article and supplemental information with more detailed information available upon request to the corresponding author.
